# Quantitative liquid-state ^1^H NMR as a tool to map phase diagrams of oleogels

**DOI:** 10.1016/j.crfs.2026.101391

**Published:** 2026-03-23

**Authors:** Senem Yilmazer, Katia Pina Chagas, Duncan Schwaller, Jean-Philippe Lamps, Bruno Vincent, Emeric Wasielewski, Michael Moir, Philippe J. Mésini

**Affiliations:** a Institut Charles Sadron, Université de Strasbourg – CNRS, 23 Rue Du Loess, Strasbourg, FR-67000, France; b Service de R.M.N, Université de Strasbourg – CNRS Fédération de Chimie Le Bel, 1, Rue Blaise Pascal, Strasbourg, 67008, France; c Plateforme RMN Cronenbourg, CNRS UMR 7042 LIMA, 25 Rue Becquerel, Strasbourg 2, FR-67087, France; d National Deuteration Facility, ANSTO, New Illawarra Rd, Lucs Heights, NSW, 2234, Australia

**Keywords:** Oleogels, Phase behavior, NMR, Triolein, Rapeseed oil, 12-Hydroxystearic acid

## Abstract

Oleogelators are compounds able to gel edible oils at concentrations of a few weight percent. They are widely studied as texturing agents for edible oils, and the resulting oleogels are considered healthier alternatives to solid fats. Understanding the phase behavior of gelator/oil, systems and mapping their *c*-*T* phase diagrams allows control of melting and formation parameters and also reveal the possible presence of polymorphs, eutectics or phase separation. The purpose of this study is to show how variable-temperature liquid-state nuclear magnetic resonance (NMR) spectroscopy can be used to map the concentration–temperature (*c*–*T*) phase diagrams of oleogels. Three gelators were selected for this investigation – DL-12-hydroxystearic acid (12-HSA), *N*-palmitoylethanolamide (Palm-EA), and *N*-palmitoyl-L-phenylalanine (Palm-Phe) – because their phase diagrams have already been reported in the literature. In addition to a classical gel–sol transition, Palm-Phe exhibits a polymorphic gel–gel transition, providing a test case to evaluate the technique. Using deuterated triolein as the solvent and a single sample per system, the method yields the liquidus line with more than 20 data points across a concentration range spanning more than one order of magnitude (typically ∼ 0.1 to ∼ 5 wt%). The liquidus lines obtained by NMR match those derived from differential scanning calorimetry (DSC), with deviations typically under 3 °C. Rheological measurements report gel-sol transition temperatures a few degrees lower than those obtained by NMR and DSC, reflecting the mechanical rather than thermodynamic nature of the transition. With Palm-Phe gels, the liquidus of the stable polymorph was measured with the same precision after annealing. Before annealing, the measured curves showed a marked difference due to gel-gel transition. The phase diagrams measured in triolein are close to those measured in rapeseed oil, indicating that triolein reproduces the phase behavior of the studied gelators in rapeseed oil.

## Highlights

• NMR in deuterated triolein maps oleogel phase diagrams from one sample.
• Diagrams include ∼20 points across more than one decade of concentration.
• NMR liquidus agrees with DSC within 3 °C for the three tested gelators.
• Triolein mimics phase behavior of rapeseed oil in oleogels.

## Graphical abstract

**Figure:**
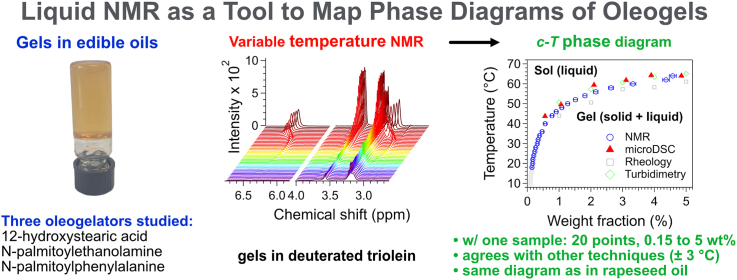

## 1 Introduction

Traditional diets, characterized by whole or minimally processed food, have given way in contemporary society to industrial and prepared food products (Poti et al., 2017). Saturated solid fats have become common ingredients in many processed foods, providing desirable organoleptic properties such as texture, mouthfeel, and flavor. However, the presence of *trans* fatty acids (Kodali, 2014) in these fats is associated with various health disorders such as cardiovascular disease (Brouwer et al., 2013; Castelli, 1986; Clifton and Keogh, 2017; Hu et al., 2001; Mozaffarian et al., 2006), inflammation, oxidative stress and metabolic syndrome (Martins et al., 2020).

In response, both consumers and health authorities are urging the food industry to reduce or eliminate these fats from food products. Conversely, vegetable oils rich in polyunsaturated fatty acids (PUFAs) offer significant health benefits, but since they are liquid they cannot play the texturing role of solid fats.

In this context, *oleogels* have emerged as promising alternatives to solid fats (Marangoni and Co, 2012; Davidovich-Pinhas et al., 2016; Marangoni and Garti, 2018; Scharfe and Flöter, 2020; Wesdorp et al., 2014). They are formed by the aggregation of low-molecular-weight compounds, known as *oleogelators*, in lipid matrices. Similarly to *organogelators* in organic solvents (Fages, 2005; Weiss, 2018; Weiss and Terech, 2006), oleogelators aggregate through self-assemblies or crystallization, driven by non-covalent interactions, such as hydrogen bonding, van der Waals interactions, or π–π stacking. The formed aggregates are typically solid-like, possess a high aspect ratio (fibers, ribbons, or platelets) and interconnect to form large clusters. At a given concentration the system percolates: the aggregates further connect to form continuous network that endows the mixture with the viscoelastic properties of a gel below its melting temperature. Oleogelators structure edible oils without altering their constituents, thereby preserving their nutritional quality (Pinto et al., 2021).

A fundamental step in processing oleogelators is understanding their phase behavior — how their formation and melting temperatures vary with concentration. This information, represented in concentration–temperature (*c*–*T*) phase diagrams, is essential for optimizing the processing conditions and predicting texture stability during storage. Such diagrams also reveal structural features such as eutectic or polymorphic transitions (Benziane et al., 2012; Costa et al., 2010; Maximo et al., 2013). These transitions transform the structure of the aggregates constituting the gel network, e.g., from a crystalline form to another (Schwaller et al., 2021b, 2023). For instance, oleogels of N-palmitoyl-L-phenylalanine (Palm-Phe) in rapeseed oil display such polymorphic transition, which reorganizes the network, from thin to thicker fibrils with distinct crystalline structures (Schwaller et al., 2023). This transformation results in a gel-gel transition, in addition to the classical gel-sol transition, and significantly alters the thermal and rheological properties, highlighting the importance of identifying such transitions in phase diagrams.

Differential scanning calorimetry (DSC) is the standard technique for constructing such diagrams; however, the number of data points is limited to the number of samples at various concentrations and reduced sensitivity for low concentrations. Low-resolution NMR is an internationally standardized method to measure accurately solid fat fractions but only above 2 wt% (AOCS, 2009; Van Putte and Van den Enden, 1974). In contrast, we have shown that liquid-state nuclear magnetic resonance (NMR) spectroscopy is a powerful alternative for mapping phase diagrams of organogelators in organic solvents. Using a single sample, variable temperature NMR spectroscopy (VT-NMR) can provide numerous data points across more than a decade of concentrations from 0.1 wt% to a few wt% (Christ et al., 2018a; Schwaller et al., 2021b). It can also detect and map monotectic or metastable solid–solid transitions. To date, however, this approach has not yet been implemented for gels in edible oils.

**Scheme 1:**
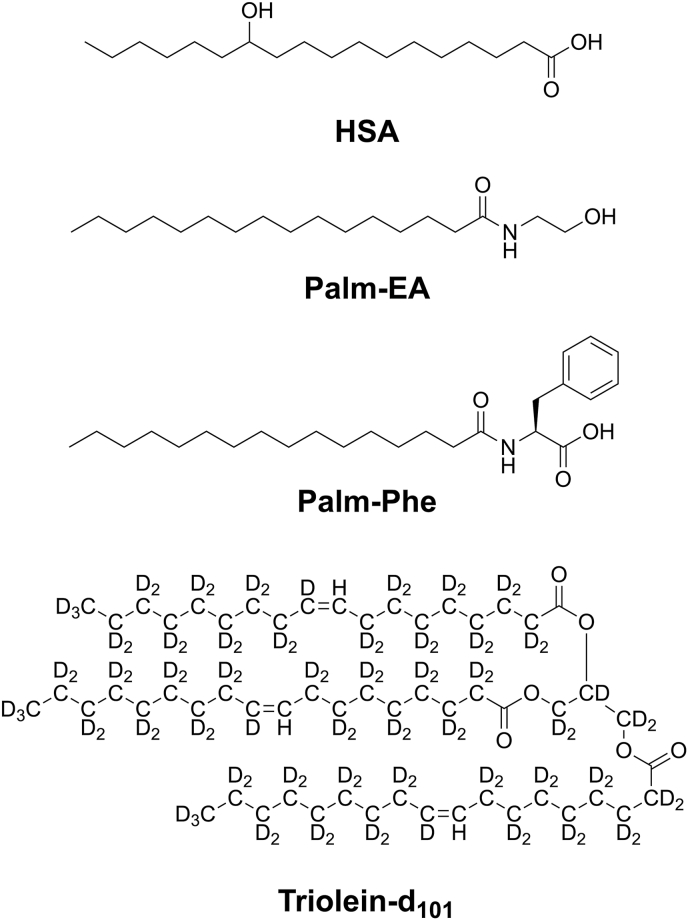
Chemical structures of oleogelators: HSA, Palm-EA, Palm-Phe, and of triolein-d_101_. Note that the vinyl C10 position is not deuterated.

In the present work, we hypothesize that triolein is a relevant model for edible unsaturated oils, and we apply liquid-state NMR to map the *c*–*T* phase diagrams of three different oleogelators in deuterated triolein (Scheme 1): N-palmitoylethanolamide (Palm-EA) (Schwaller et al., 2022), N-palmitoyl-L-phenylalanine (Palm-Phe) (Schwaller et al., 2023) and DL-12-hydroxystearic acid (12-HSA) (Elliger et al., 1972; Rogers et al., 2009a). These compounds were selected because their phase diagrams have previously been established in rapeseed oil (Schwaller et al., 2022, 2023) or canola oil (Rogers et al., 2009b) allowing direct comparison between their phase behavior in natural oils and in triolein. They also have been selected for complementary reasons. 12-HSA is well-studied model organogelator (Terech et al., 1994) and oleogelator (Rogers et al., 2008, 2009c); Palm-EA is an endogenous fatty amide with reported nutraceutical benefits (Clayton et al., 2021); and Palm-Phe exhibits polymorphism in oil, constituting a test case to evaluate the ability of NMR to detect such transition. While previously applied to organogelators in organic solvents, this study is the first systematic implementation in oil systems. The present work demonstrates that (i) NMR enables high-resolution mapping of oleogel phase diagrams with more than twenty data points from a single sample, (ii) the method is validated by comparing NMR-derived phase diagrams with those established by other techniques (iii) the measurements reveal marked differences before and after annealing for an oleogel with polymorphic transitions, (iv) triolein reproduces the phase behavior of the studied oleogels in rapeseed oil.

## 2 Materials and methods

### 2.1 Materials

Palm-EA and Palm-Phe were prepared according to published procedures (Schwaller et al., 2022, 2023). 12-HSA (CAS 106-14-9, racemic mixture), triolein (glycerol trioleate, CAS 122-32-7) and bis-(trimethylsilyl)acetylene (BTMSA) (CAS 14630-40-1) were purchased from TCI Chemicals. 12-HSA contains non-hydroxylated fatty acids as impurities. It was purified by recrystallization from ethanol, followed by Soxhlet extraction with petroleum ether (Bawn and Huglin, 1962). Purity was confirmed by thin-layer chromatography (TLC), NMR and elemental analysis (found: C% 71.95; H% 12.09; calcd. for C_18_H_36_O_3_: C% 71.95; H% 12.08). The rapeseed oil, purchased from a local store (Bouton d’Or, Intermarché) is refined and contains more than 99 wt% of triacylglycerol. Its composition in fatty acids is approximately 92% unsaturated acids (64% monounsaturated, 10% α-linolenate, 8% linoleate) and 7% saturated acids. Triolein-d_101_ was synthesized by the National Deuteration Facility, Australian Nuclear Science and Technology Organisation (ANSTO), via a Steglich esterification of oleic acid-d_32_ (Darwish et al., 2013) with glycerol-d_5_ (**Suppl. Info.**). Its structure is shown in Scheme 1.

Triolein-d_101_ is not fully deuterated, as the vinyl protons at the C10 positions of the oleoyl groups remain. The isotopic purity was measured to be 91% (**Suppl. Info.**). The deuteration level is reduced by partial back-exchange at the C2 position of the oleoyl chains.

### 2.2 Preparation of the gels and parameters

The gels were prepared by accurately weighing the oleogelator and triolein according to the targeted mass fraction *W* (wt%). The resulting mixtures were heated until complete dissolution and the gels were formed by cooling the solutions *in situ* in the measuring cells or tubes for each technique (see sections 2.3, 2.4, 2.5, 2.6 for specific protocols). For rheology, microDSC and turbidimetry, the samples were all prepared at the same cooling rate (0.25 °C/min) and the transition temperatures were measured with the same heating rates in order to facilitate direct comparisons. NMR employs a step heating ramp, resulting in a slower average rate of (0.087 °C/min).

For rheology and DSC, protiated H-triolein was used, while for NMR, deuterated D-triolein was used. As D- and H-triolein differ in molar mass, weight fractions in D-solvent *w*_D_ were converted to their equivalent mass fraction in H solvent *w*_H_, according to:

Eq.(1) $$w_{H}=\frac{w_{D}r}{1+w_{D}\left(r-1\right)}$$

with *r* = *M*_D_/*M*_H_, the ratio of the molar mass of the D-triolein to that of the H-triolein (*r* = 1.115). Solutions in D- and H-triolein of respective concentrations *w*_D_ and *w*_H_ (as calculated by Eq. (1)) have the same molar fraction. For example, a Palm-EA concentration in triolein-d_101_ *W*_D_ = 4.00%, is equivalent to a concentration in H-triolein *W*_H_ = 4.44%.

The melting temperatures of the pure gelators, were determined by DSC through linear extrapolation at a null heating rate: PalmEA: 98.8 °C, 12-HSA: 83.7 °C, PalmPhe: 91.9 °C. The maximal melting temperatures of the studied gelator/triolein mixtures were found by NMR and DSC: PalmEA-4.44 wt%: 89.4 °C, HSA-4.98 wt%: 64.0 °C, PalmPhe 2 wt% 54.4 °C, PalmPhe-5 wt%: 63.6 °C.

### 2.3 Rheology

The elastic modulus *G′* and the viscous modulus *G″* were measured with a stress-controlled rheometer (Mars III, Haake) equipped with a double Couette cell. The device applies a controlled stress and measures the resulting strain transmitted through the sample. Temperature was controlled by a heated bath with a precision of ±0.05 °C. The samples were prepared by weighing the gelator and the oil in a vial, sealing it with a Teflon-liner stopper and heating the mixture at 80 °C until complete dissolution. The gels were formed *in situ* by pipetting 7 mL of this hot solution into the cell, then cooling to 20 °C at – 0.25 °C/min. At 20 °C, a frequency sweep at 0.1 Pa was performed to verify that *G′* was constant and *G′* > *G″* (Fig. S1, Suppl. Info). A stress sweep at 1 Hz determined the linear viscoelastic regime (LVR, Fig. S2, Suppl. Info). *G*′ and *G″* were measured at 1 Hz, with a stress within the LVR, from 20 to 80 °C with heating rate of 0.25 °C/min (Fig. S3, Suppl. Info). The temperature of the gel-sol transition *T*_GS_ was defined as the temperature at which *G′* and *G″* intersect.

### 2.4 Micro differential scanning calorimetry (μ-DSC)

The thermograms were recorded with a μ-DSC (MC-DSC, TA instruments) with three independent sample cells (gelator/triolein gels at different concentration) and a reference cell (triolein only). The gelator and the oil were accurately weighed in the measuring cells (resp. precision ±20 μg and 1 mg) to reach the targeted weight concentrations *W* (0.5 – 5 wt%). The final weight of the samples was approximately 500 mg. The total weights (cell + sample + cap) were carefully balanced within 5 mg. The thermal cycle consisted of the following steps: 1) heating samples above their melting temperature 2) cooling to 5 or – 10 °C at – 0.25 °C/min; 3) resting at low temperature for 1 h; 4) reheating at 0.25 °C/min. The gel was formed during step 1 and 2, and the showed thermogram measured during step 4. The temperature ranges were 5 °C to 110 °C for Palm-EA, −10 °C to 95 °C for 12-HSA, and −10 °C to 80 °C for Palm-Phe. The melting temperatures were determined as the inflection point after the maximum of the endotherm peak, as described and justified in our previous work (Schwaller et al., 2021a).

### 2.5 Turbidimetry

The turbidity of Palm-EA/triolein and Palm-Phe/triolein mixtures were measured as a function of temperature using a home-made device described in detail elsewhere (Schwaller et al., 2022). The setup consists of passing a laser beam (He–Ne, *λ* = 632.8 nm) through the sample (optical path length 5 mm), and to record the transmitted intensity with a CCD camera. The gels were formed in the measuring cell, by loading 2 mL solution (80 °C fully dissolved) cooling it from 80 to 20 °C at a rate of −0.25 °C/min and letting it rest 30 min. Transmitted intensity was measured, while the sample was heated from 20 to 80 °C at a constant rate of 0.25 °C/min. This method relies on light scattering by gel network aggregates. Upon heating, these scattering entities dissolve and transmittance increases sharply. The onset temperature for which transmittance reaches its maximum was identified as the transition temperature and reported in the phase diagrams for Palm-EA/triolein and Palm-Phe/triolein systems. For the 12-HSA/triolein system, direct turbidimetry proved insufficiently sensitive due to weak scattering. Therefore, absorbance was monitored with a JASCO V770 spectrometer at 550 nm, a wavelength where neither component absorbs. The transition temperature was defined as the temperature at which the absorbance dropped to zero.

### 2.6 NMR experiments

**Fig. 1:**
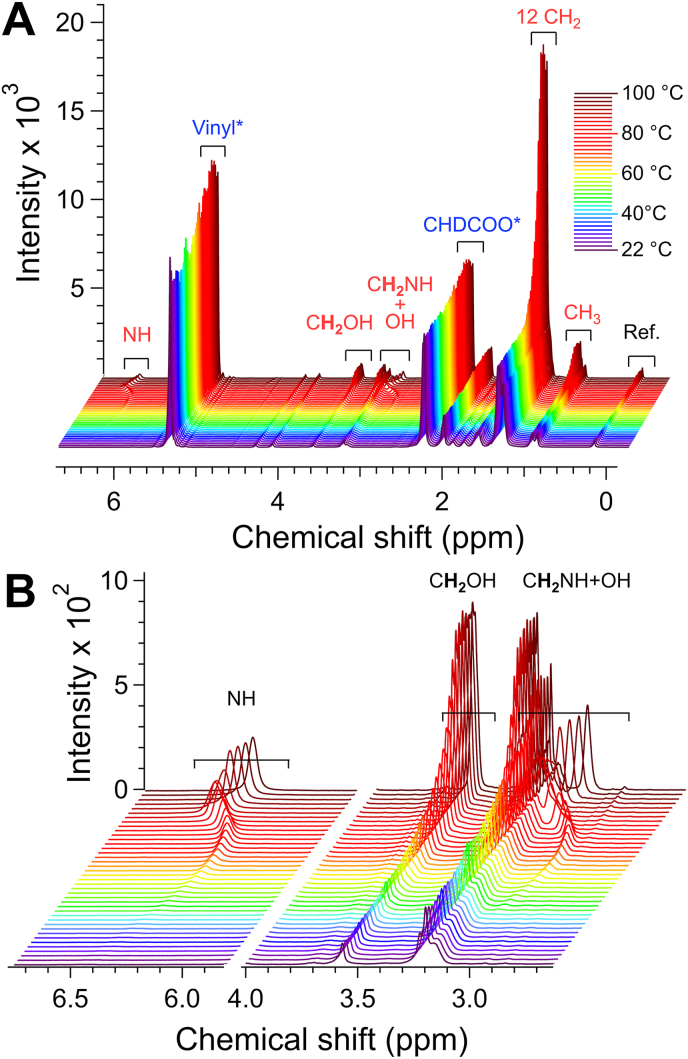
NMR spectra of Palm-EA in triolein-d_101_ (*W*_H_ = 4.44 wt%) from 22 °C to 100 ° C. A: full spectrum data with assignments for Palm-EA (complete peak assignments in Suppl. Info.). ∗: peaks of triolein. **B:** expanded view of the 6.8–5.2 and 4–2 ppm regions.

The NMR spectra were recorded at various temperatures on a Bruker Avance III HD 400 MHz (Palm-EA, Palm-Phe) or Bruker Avance III 600 MHz (12-HSA). Temperature was calibrated with 80% ethylene glycol (DMSO-d_6_) for high temperatures (293 K to 380 K). For low temperatures (203 to 298 K), calibration was carried out with 4% methanol in methanol-d_4_ for the 400 MHz spectrometer (source: Bruker Instruments, Inc. VT-Calibration Manual, accuracy ±0.2 °C) and with 99.8% methanol-d_4_ (Karschin et al., 2022) for the 600 MHz spectrometer. The samples were prepared by heating the gelator (weighed with a precision of ±20 μg), triolein-d_101_ and about 1 % of BTMSA at 100 ± 1 °C until complete dissolution. Then, 0.5 ± 0.05 g of the solution was transferred while hot into NMR tubes and cooled to 20 °C (12-HSA) or 10 °C (PalmPhe) at a rate of −0.1 °C/min and lest rest 1 h before measurements. For Palm-Phe, samples with two different thermal treatments were prepared: 1) cooling rapidly the hot solution in a bath at – 10 °C and kept below 0 °C less than 1 h before the measurements. 2) annealing at 40 °C for 7 h and cooling back at – 10 °C (for two samples at 2 and 5 wt%). At 40 °C the samples remained in a gel state. During the measurements, temperature was incremented by steps of 2 °C. After each increment, the sample was equilibrated 7 min after temperature stabilization before recording the next spectrum. Spectra were recorded with a zg30 pulse sequence, 32 scans and the relaxation delay of 30 s. The overall heating rate was 0.087 °C/min. The temperature ranges were 10–80 °C (Palm-EA), 20–80 °C (12-HSA) and 0–100 °C (Palm-Phe). These ranges were chosen to cover all transitions observed with the other techniques while remaining narrow enough to limit the duration of the NMR experiments. The spectra were processed using MestReNova (MestreLab research), with BTMSA peak at 0.144 ppm as a reference. The integrations were performed with Igor Pro 9 (Wavemetrics). Fig. 1 shows the spectra obtained for Palm-EA (*W_H_ =* 4.44 wt %).

### 2.7 NMR data normalization and corrections

**Fig. 2:**
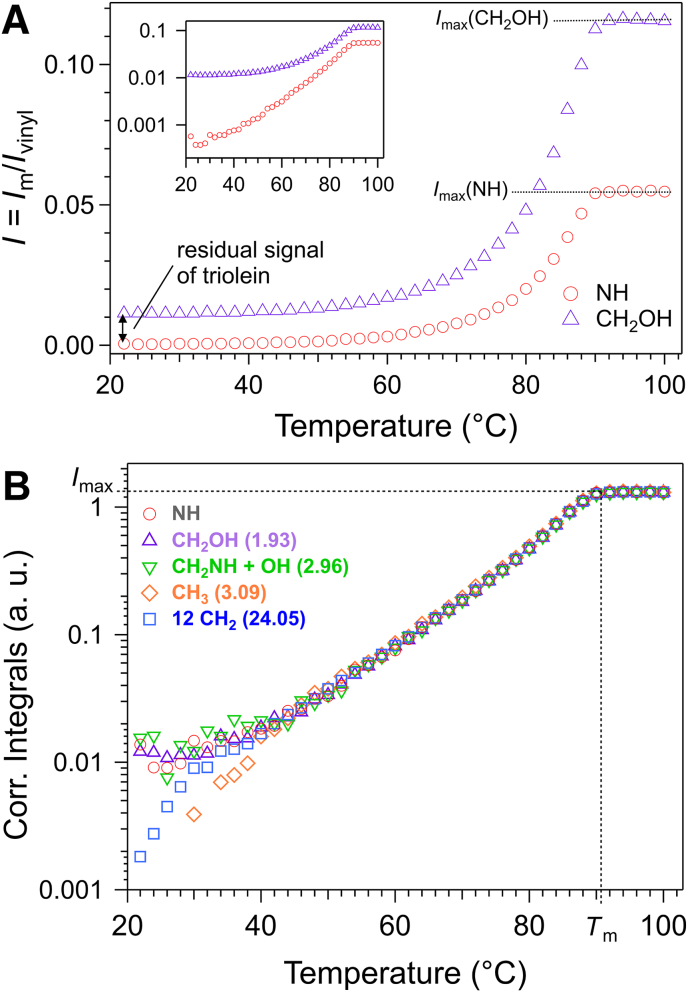
A: Integrals of NH and CH_2_OH (normalized to the integral of the vinyl proton of triolein). Inset: semi-logarithmic representation. The CH_2_OH signal overlaps with peaks of oil, which results in a gap with the integral of NH at low temperatures. **B**: normalized integrals after correction from oil signals. For clarity, the curves have been multiplied to superimpose *I*_max_ of the different peaks. The numbers in parentheses are the found multiplicity after correction (*I*_max_/*I*_max_(NH)). *T*_m_ is the temperature at which the intensities reach their maximum *I*_max_ and corresponds to the complete melting of the solid phase.

The different steps of the data treatment are explained with the example of Palm-EA. The integrals of the signals of triolein (e.g. the vinyl signal at 5.23–5.40 ppm, Fig. 1A) and reference (0.14 ppm) show a typical and parallel evolution with temperature (Fig. S4, Suppl. Info), which verifies that the reference compound does not interact with the oil. The integrals of gelator signals were normalized against the integrals of BTMSA signals or against those of the vinyl signal of triolein-d_101_ (Fig. S4, Suppl. Info.). The normalized intensities *I* reach a plateau (value *I*_max_), after a temperature *T*_m_ corresponding to the complete dissolution of the gelator at the nominal concentration *W* of the sample. (Fig. 2A). *I*_max_ was measured after averaging the plateau (the associated error estimated as the standard deviation).

For pure gelator signals (such as NH in Palm-EA, between 6 and 6.5 ppm, Fig. 1B), the integrals drop to near zero at low temperatures. The plateau *I*_max_(NH) corresponds exclusively to the gelator and integrates to 1 H (Fig. 2A). For other gelator signals (e.g. C**H**_2_OH) overlap with triolein leads to a ‘floor’ integral *δ* at low temperatures, and an excess value of the plateau with the same value *δ* = *I*_max_(C**H**_2_OH)-2*I*_max_(NH). *δ* is subtracted from the integrals of C**H**_2_OH. After this first raw correction, the integrals of different protons overlap within scaling factors proportional to the proton counts for temperatures above 50 °C (Fig. 2B). At lower temperatures, due to the limited precision (±2%) in determining plateau values, residual baseline offsets or negative values may persist. The *δ* values were therefore fine-tuned within a range of ±1 to ±4% to eliminate these artifacts and align the corrected intensities with the NH signal at all temperatures. Notably, the corrected curves remain well-aligned down to 40 °C (Fig. 2B). This adjustment accounts for minor discrepancies in proton multiplicity at the plateau, as shown in Fig. 2B.

## 3 Results and discussion

### 3.1 Measurement of the spectra, normalization and correction of the integrals

We have measured the NMR spectra of the three studied systems, while increasing temperature. The sample concentrations were chosen between 4.4 and 5 wt% in order to explore the range of concentrations practically used in applications, as the selected oleogelators provide sufficient texturing for at concentrations below 5 wt%. In this section, we summarize how the integrals of the gelator peaks were normalized and corrected to collect the quantitative information necessary for mapping the diagrams.

In all spectra, the most intense signals arise from triolein-d_101_, as seen in the 4 wt% Palm-EA/triolein sample (Fig. 1A). These include the C-10 vinyl protons of the triolein-d_101_ at 5.3 ppm and other residual signals due to isotopic impurities (e.g. CHD-COO at 2.21 ppm). The signals of the gelator are one order of magnitude smaller. After normalization of the spectra to a reference or to triolein vinyl signal, the integrals of the gelator peaks increase with *T* and reach a plateau at the temperature *T*_m_. Above *T*_m_ the gelator is fully dissolved, and the normalized integrals of its signals remain constant. Most of the gelator signals overlap with small signals from triolein, but as described in section 2.7, this contribution can be corrected by considering the residual intensities at low temperature and the excess values of the plateau. The normalized and corrected intensities (Fig. 2) superimpose above 40 °C with scaling factors proportional to the proton multiplicity. At lower temperatures the curves are dispersed due to higher errors in the measurement of the integrals. These corrected integrals were used to derive the phase diagrams as explained in the following section.

### 3.2 Interpretation of the curves, conversion to concentration and to phase diagram

As established in section 3.1, the plateau regions correspond to full dissolution of the gelator. The normalized integral, *I*_max_ is then directly proportional to the total gelator concentration *W*, i.e., *I*_max_ ∼ *W*.

**Fig. 3:**
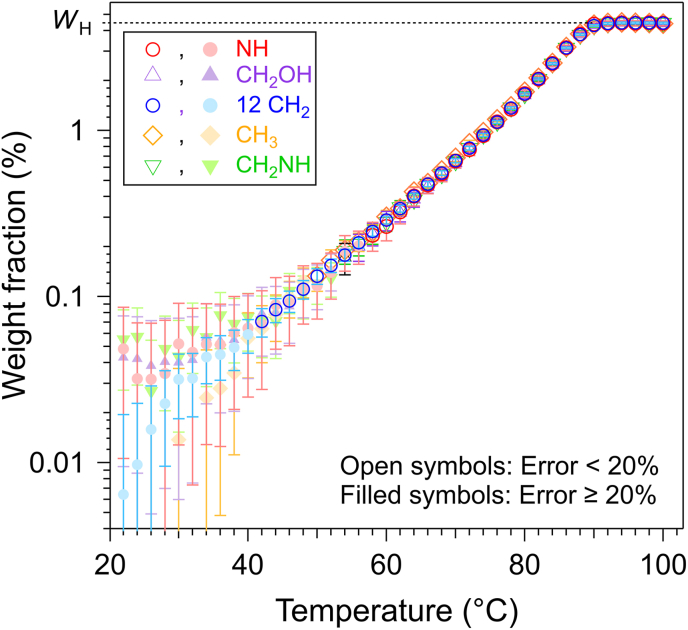
Weight fractions calculated from different peaks of PalmEA. *W*_H_ is the total concentration of the gelator in the sample.

Below *T*_m_, the network of the gelator is silent in liquid-state NMR due to its large solid-like structure and restricted mobility, which broaden its signals beyond detection limits. Thus, liquid-state NMR detects only the soluble fraction of the gelator (Hirst et al., 2009; Wallace et al., 2015; Morris et al., 2013; Edwards and Smith, 2013; Frkanec et al., 2002; Cornwell et al., 2015; Ardoña et al., 2017; Escuder et al., 2006). As temperature increases, the solid fraction partially melts and is dissolved in the oil, resulting in an increase of the fraction *w*(*T*) of the gelator in solubilized in oil. *w*(*T*) increases until it reaches the total concentration (*w*(*T*) = *W*). Since the integrals *I* are proportional to the soluble fraction, *w*(*T*) is calculated using the following equation:

Eq.(2) $$w\left(T\right)=W\frac{I}{I_{\max}}$$

where *I* is the normalized integrals, *I*_max_ the value of the plateau and *W* is the total weight fraction. Since NMR is conducted in D-solvent, *w*(*T*) and *W* represent weight fraction in D-triolein. In order to compare with other techniques, they converted into the equivalent concentration in H-triolein using Eq. (1) or by substituting *W* by *W*_H_ in Eq. (2). In summary, all gelator signal integrals were normalized and corrected for solvent overlap, then converted into weight fractions using Eqs. (1) and (2). This process was repeated for the different signals of the gelator (Fig. 3).

Above 40 °C, the concentrations calculated from the different proton signals agree within ±4%. Below this temperature, they begin to diverge due to increased experimental uncertainty. The uncertainty in *w*(*T*) was calculated by accounting for integration errors, limited integration range (∼2%), weighing inaccuracies (∼0.5%), and the standard deviation of the *I*_max_ values (∼2%). Integration errors arise primarily from the signal-to-noise ratio (SNR): at high temperatures, where SNR is high, these errors remain below 1%. However, at low temperatures, the decreased SNR can lead to integration errors exceeding 50%. In Fig. 3, data points with errors below 20% are distinguished (open symbols) and are considered reliable. They are found above 40 °C and at concentrations greater than 0.07 wt%, which indicates the sensitivity limit of our method.

We have verified that the integrals of various signals superimpose, which is expected for pure organogelators. However, for food applications, many gelators are mixtures, such as natural waxes which composed of long alkanes and fatty esters (Alvarez-Mitre et al., 2013; Blake et al., 2014; Dassanayake et al., 2009; Hwang et al., 2012; Jana and Martini, 2014). For such mixtures, the integrals will superimpose if all the constituents have equivalent solubilities as a function of *T*. A similar analytical approach could be applied for these mixtures either to confirm uniform solubility behavior or, in case of significant deviations, quantify differential solubilities between mixture components.

### 3.3 Phase diagrams from the NMR

**Fig. 4:**
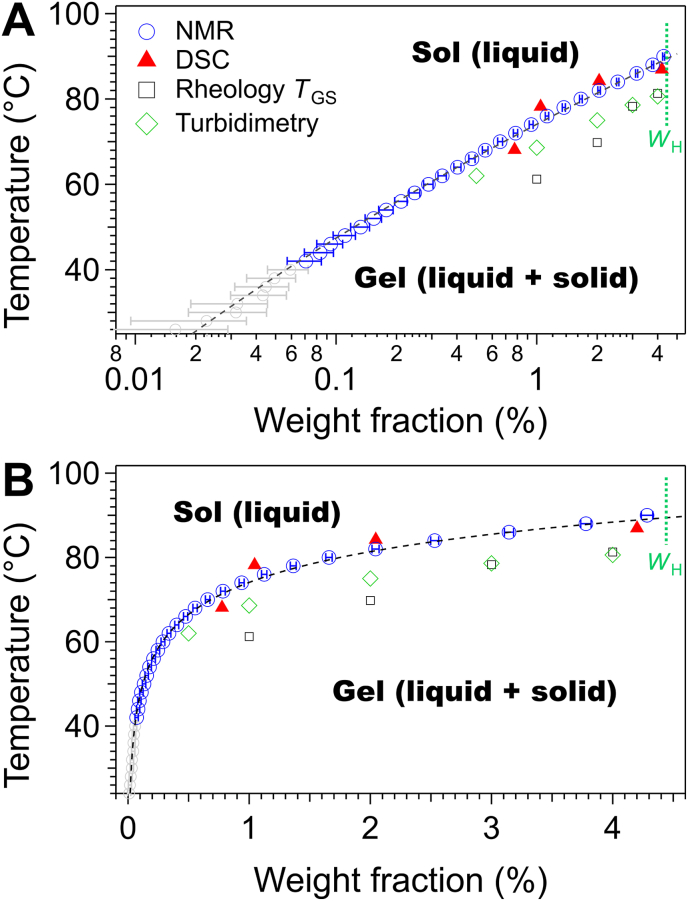
*c*-*T* phase diagram of Palm-EA/triolein in semilog (A) and B: linear (B) representation. The concentrations from NMR correspond to the peak 12 CH_2_ and its corrected integral (Fig. 2, Fig. 3). For NMR, the points with uncertainty >20% have been represented in light grey. *W*_H_: total gelator concentration in the sample.

As established in previous sections, the *w*(*T*) values below *T*_m_ represent the concentration of the soluble fraction, so by definition, the liquidus line of the *c*-*T* phase diagram. Above *T*_m_ the gelator is fully dissolved, the system is no longer in phase equilibrium and the plateau no longer represents the liquidus. One curve (from the 12CH_2_ peak), is plotted in Fig. 4 as a classical phase diagram, after switching the axes (*T* vs. concentration), both in semi-logarithmic (A) and linear (B) representations. Two regions are indicated: below 40 °C, the data with high uncertainty displayed in light grey (excluded from analysis); between 40 and 90 °C, the liquidus points with acceptable precision (useable liquidus data). The points of the plateau (above 90 °C) are replaced by a line indicating the total concentration *W*_H_ of the sample.

The useable liquidus data derived from NMR correspond to concentrations comprised between 0.07 and 4.44 wt%. This demonstrates that NMR is one order of magnitude more sensitive than conventional techniques, with threshold concentrations of 0.8 wt% for microDSC and 0.5 wt% for rheology. The NMR derived liquidus aligns with the values from DSC within an experimental error of 3.5 °C, which is consistent with errors commonly observed in such systems (Terech et al., 2000; Toro-Vazquez and Pérez-Martínez, 2018). The gel-sol temperatures *T*_GS_ determined by rheology are systematically lower than those from DSC, NMR and turbidimetry, as already observed in rapeseed oil (Schwaller et al., 2022). This reflects the physical nature of the transitions: NMR and DSC measure the temperature *T*_m_ where all solid has melted (thermodynamic). Rheology measures the temperature *T*_GS_ where the particles are still present but lose network connectivity (mechanical). The gap between the values by NMR and rheology widens at low concentration. For example at 1 wt%, *T*_GS_ = 61.2 °C and *T*_m_ = 74 °C. A similar gap is observed in rapeseed oil (Schwaller et al., 2022) and in olive and cosmetic oils (Chen et al., 2026), showing the generality of this phenomenon across different oils. The difference between DSC and rheology cannot be explained by a difference in the heating/cooling rates since they are the same. Between *T*_GS_ and *T*_m_, a large fraction of the solid aggregates has not melted and the sample is a suspension with a liquid behavior. As shown previously by SEM and XRD (Schwaller et al., 2022), the aggregates are rather flat, with a lamellar structure and low aspect ratio, which reduces their ability to percolate at low concentration. The values measured by turbidimetry lie between NMR and DSC, confirming the behavior observed in rapeseed oil. For technical reasons, the heating rate employed for NMR (0.087 °C/min) is slower than for the other techniques (0.25 °C/min). However, as shown in previous work (Christ et al., 2018b; Schwaller et al., 2021b), when heating rates are sufficiently slow, they do not affect the measured phase transition temperatures.

### 3.4 Phase diagram of other oleogels

**Fig. 5:**
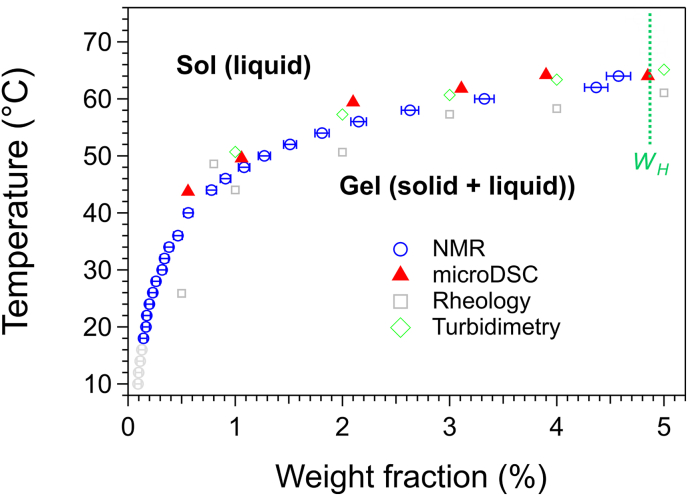
*c*-*T* Phase diagram of 12-HSA/triolein. For the NMR data, the points with uncertainty >20% have been represented in light grey (below 18 °C). *W*_H_: total gelator concentration in the sample.

The method described for Palm-EA/triolein was also applied to establish the phase diagram of 12-HSA in triolein (Fig. 5). NMR spectra were measured for a 5 wt% gel between 10 and 80 °C. The normalized integral of the CHOH peak at 3.5 ppm was used to determine the liquidus line. The points below 18 °C exhibit relative errors >20% and are displayed in light grey. Therefore, the practical range of measured concentrations spans form 0.15 to 5 wt%. The liquidus temperatures determined by NMR agree with those measured by DSC and turbidimetry, within a margin of ±2.5 °C, showing a good agreement between these techniques.

Rheological measurements again yielded gel-sol transition temperatures *T*_GS_ lower than the *T*_m_ values obtained from NMR and DSC (by ∼2-4 °C), consistent with the fact that rheology detects the loss of mechanical integrity before the full melting of the network particles. At concentrations below 0.5 wt%, the samples are no longer gels and there are no gel-sol transitions. Nevertheless, NMR can still detects phase transitions, corresponding to the melting solid particles forming a liquid suspension, but no solid connected network.

**Fig. 6:**
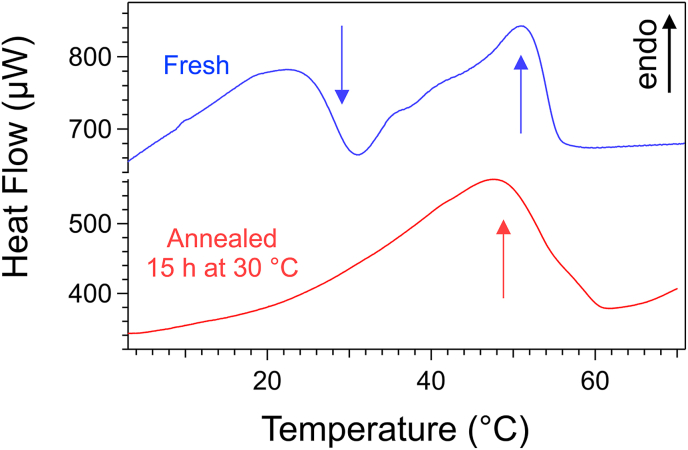
Thermograms of gels of Palm-Phe in triolein (3 wt%) subjected to different thermal treatments. Both gels were formed by cooling at – 0.25 °C/min and maintained at 0 °C for 1 h. The thermogram of the fresh gel was recorded immediately afterward. The thermogram of the annealed gel was recorded after an additional heating from 0 to 30 °C, an isotherm at 30 °C for 15 h and cooling to 0 °C. Upward Arrow: endotherm of gel melting; downward arrow : polymorphic transition.

We now evaluate the NMR method with an oloegelator exhibiting more complex phase behavior. As discussed in section 3.1, Palm-Phe exhibits in rapeseed oil polymorphism of its solid fraction, resulting in a gel-gel transition (Schwaller et al., 2023). The polymorphic transition occurs at a fixed temperature (∼27 °C), independent of concentration, and a separate gel-sol transition occurs at higher temperatures, varying with concentration. The same behavior is observed in triolein. DSC thermograms show two transitions for freshly prepared samples (Fig. 6, top curve): at ∼22 °C a gel-gel transition (polymorph transformation); at ∼50 °C, an endotherm corresponding to the melting of the second polymorph. Rheology also detects this transition via a subtle change of slope in *G′*(*T*) and a minimum in *G*″(*T*) (Fig. S5, Suppl. Info).

**Fig. 7:**
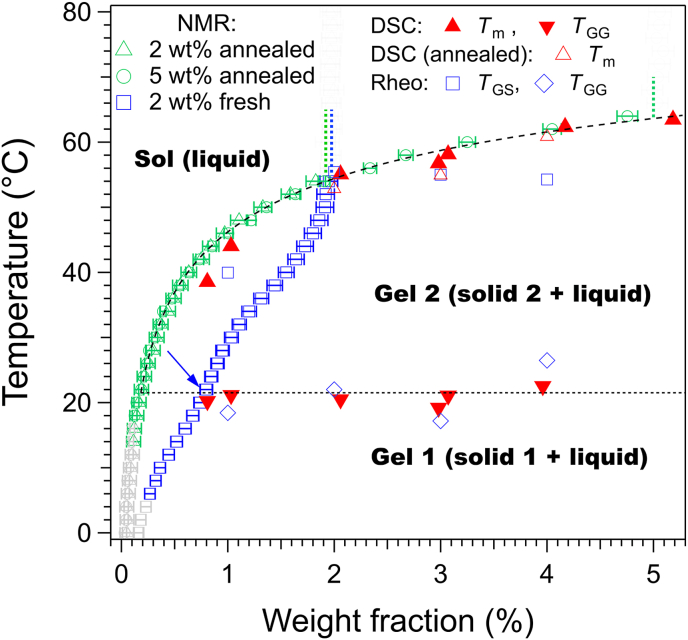
*c*-*T* Phase diagram of Palm-Phe/triolein mapped by NMR, DSC and rheology. Fresh samples: prepared by rapid cooling and maintained at 0 °C less than 1 h before measurement; annealed samples: annealed at 40 °C for 7 h and cooled prior to measurements. Dashed vertical lines: total gelator concentration *W*_H_ in the samples. Arrow: corner point indicating the gel-gel transition.

The gel-gel transition is observed only when the gel is freshly prepared and disappears when after annealing at 30 °C. (Fig. 6, bottom curve). We have verified for different concentrations that the *T*_m_ values measured by DSC for fresh and annealed gels agree within ±3 °C. Furthermore, the polymorphic transition disappears also when the gel is aged for one day, below the transition temperature. It shows that the polymorph constituting the fresh gels is metastable; and irreversibly transforms into the stable polymorph, even at low temperatures. The gel-gel transition is non variant: its temperature remains constant regardless the concentration (Fig. 7), in accordance with Gibbs's Phase rule for an equilibrium between two solid phases and a liquid. In summary, the polymorphism observed in rapeseed oil is reproduced in triolein, at a slightly different temperature (22 °C vs. 27 °C). The nature of the dominant polymorph depends strongly on the thermal history of the gels.

To assess the influence of polymorphism by NMR, we have studied Palm-Phe/triolein samples subjected to two different thermal treatments (Fig. 7). Annealed samples: heated at 40 °C for 7 h, and cooled before measurements, in order to map the phase diagram of the stable polymorph. Fresh samples: prepared by fast cooling and measured within 1 h.

The integrals of the gelator signals were processed as described in Section 3.1, 3.2. For the annealed samples, two concentrations were used (2 and 5 wt%). The concentration-temperature curves for both samples superimpose below 2 wt%, confirming that only the soluble fraction is NMR visible, regardless of total gelator concentration, as already reported (Christ et al., 2018a). Again, the curves align with DSC melting points, while the rheological *T*_GS_ values remain lower.

For fresh samples, only the plateau region matches with the annealed sample. Below this region, they differ markedly. As we have shown above, the fresh sample contains mainly gel 1 that undergoes a polymorphic transition upon heating, while the annealed sample contains gel 2. The difference between both curves measured from NMR confirm the difference of phase behavior between both. The gel-gel transition occurs near 22 °C as shown by DSC and rheology (horizontal dotted line in Fig. 7). The concentrations measured by NMR show a corner point at this temperature, which could be used to determine the transition temperature. However, it is less pronounced than for other systems experiencing a polymorphic transition and showing a sharp discontinuity at the transition temperature (Schwaller et al., 2021b). In the present case, the variations are attenuated, probably due to a partial or gradual transformation even below transition temperature, to the coexistence of both polymorphs, or to kinetics effects. However, NMR clearly reveals strong differences between fresh and annealed samples. These differences highlight the substantial impact of sample preparation and thermal history on the thermodynamic and structural properties of the gels.

### 3.5 Comparison between rapeseed oil and triolein

**Fig. 8:**
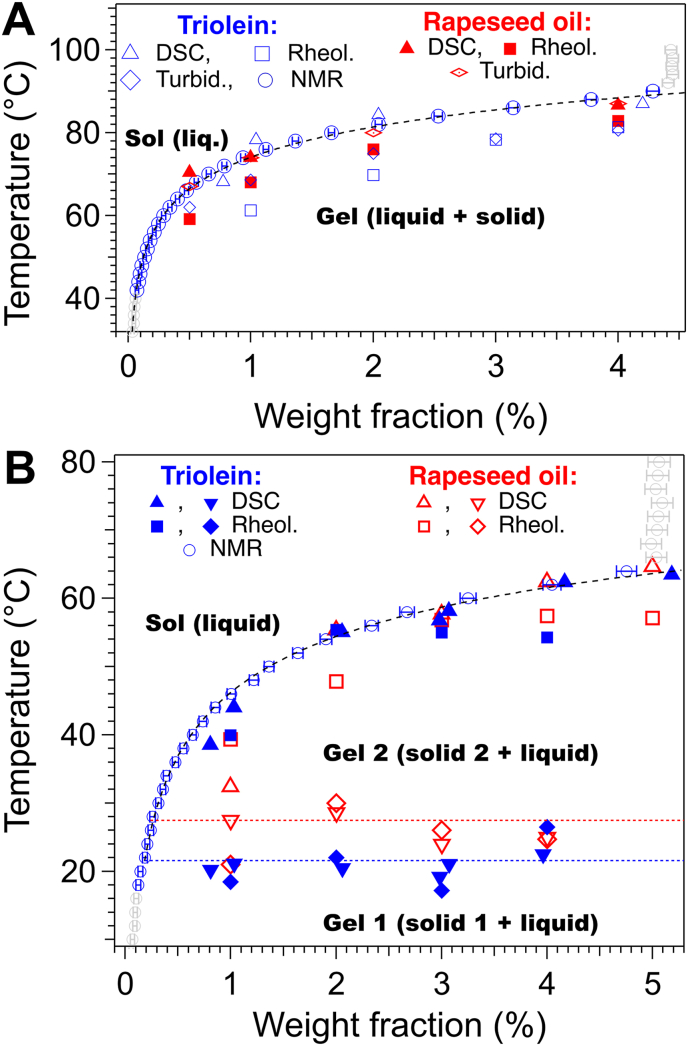
Comparison of phase diagrams in triolein and rapeseed oil. A: Palm-EA; B: Palm-Phe.

In this section, we address whether the phase diagrams obtained in triolein reflect those in rapeseed oil. The phase diagrams of Palm-EA and Palm-Phe in rapeseed oil have been previously established by DSC, rheology and turbidimetry (Schwaller et al., 2022, 2023). The phase diagrams in both oils have the same aspect (Fig. 8).

In more detail, the liquidus from DSC and NMR in both oils superimpose within 2 °C; the rheological transitions *T*_GS_ for Palm-EA have slightly higher values in rapeseed oil than in triolein and the differences vary from 2 to 7 °C, with the maximum value corresponding to the lowest concentrations. For Palm-Phe, *T*_GS_ values in rapeseed oil and triolein match within 3 °C. Finally, the gel-gel transition in Palm-Phe occurs 5 °C lower in triolein. These comparisons show that the nature of the oil does not change the positions of the liquidus, which correspond to a thermodynamical equilibrium. However, it slightly impacts the temperature of polymorphic transition. This suggests that the oil interacts with the metastable polymorphs, (through different solubility, wettability or interfacial energies) but almost not with the stable one. Since the low-temperature polymorph is metastable, the difference in *T*_GG_ may be attributed to both thermodynamics and kinetic factors (lower activation energies, nucleation …). Despite the slight difference in *T*_GG_ values, triolein proved a valid model solvent for studying the phase behavior of the proposed oleogelators in rapeseed oil.

## 4 Conclusions

Liquid-state NMR proves to be an effective technique to map phase diagrams of oleogels. This technique enables the precise determination of the liquidus line over more than one order of magnitude in concentration, with a relative uncertainty of less than 20% in the measured concentrations. The liquidus temperatures derived from NMR match those obtained by DSC within ±3.5 °C. From a food science perspective, this approach provides a powerful complementary tool to DSC for rapidly screening oleogelators and processing conditions relevant to fat replacement strategies.

Our experiments also show that the method is sensitive to gel-gel transitions and highlighted significant differences between fresh and annealed samples. However, these transitions are more clearly observed and characterized with DSC, which also offers greater control over thermal treatments such as annealing.

The liquidus of the phase diagrams obtained in triolein agree within differences of 2 °C with those established in rapeseed oil using conventional methods (DSC, rheology and turbidimetry). These findings validate triolein as a suitable model solvent for rapeseed oil, effectively reproducing the phase behavior of the selected oleogelators.

## CRediT authorship contribution statement

S. Y.: investigation, writing – original draft – review & editing, visualization; K. P. C.: investigation, writing – original draft – review & editing, visualization; D. S.: investigation; J.-P. L.: investigation; B. V.: investigation; E. W.: investigation; M. M: editing, resources; P. J. M.: conceptualization, writing – review & editing, visualization, funding acquisition, project administration.

## Declaration of competing interest

The authors declare that they have no known competing financial interests or personal relationships that could have appeared to influence the work reported in this paper.
