# Supplementary material for: Quantitative liquid-state 1H NMR as a tool to map phase diagrams of oleogels
Source: Curr Res Food Sci. 2026 Mar 23;12:101391. doi: 10.1016/j.crfs.2026.101391 (PMC13084719; doi:10.1016/j.crfs.2026.101391)
Supplement: Multimedia component 1 [file mmc1.docx]

Quantitative Liquid-state ^1^H NMR as a Tool to Map Phase Diagrams of Oleogels

**Supplementary information**

**SenemYilmazer^a,#^, Katia Pina Chagas^a,#^, Duncan Schwaller^a^, Jean-Philippe Lamps^a^, Bruno Vincent^b^, Emeric Wasielewski ^c^, Michael Moir^d^, Tamim A. Darwish^d^, Philippe J. Mésini^a,*^**

^a^Institut Charles Sadron, Université de Strasbourg – CNRS, 23 rue du Loess, FR-67000 Strasbourg, France

^b^Service de R.M.N, Université de Strasbourg – CNRS Fédération de Chimie Le Bel, 1, rue Blaise Pascal, 67008 Strasbourg, France

^c^Plateforme RMN Cronenbourg, CNRS UMR 7042 LIMA, 25 rue Becquerel, FR-67087 Strasbourg 2, France

^d^National Deuteration Facility, ANSTO, New Illawarra Rd, Lucs Heights NSW 2234, Australia

^#^these authors contributed equally

**Synthesis of triolein-d_101_**

| 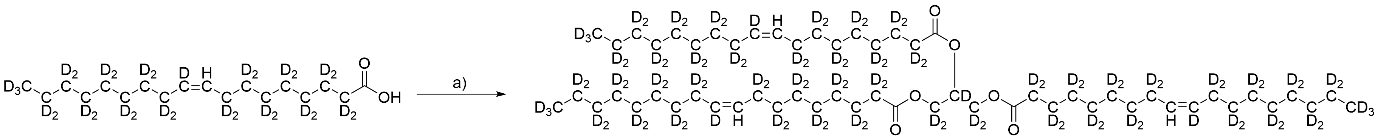 |
| --- |
| **Figure a.** Synthesis of triolein-d_101_. Reagents and conditions: (a) Glycerol-d_5_, DMAP, DCC, CH_2_Cl_2_, 24 h, rt, 75%. |

*General Experimental*

All reactions were performed under an atmosphere of nitrogen unless otherwise specified. Chemicals and reagents of the highest grade were purchased from Sigma-Aldrich (Sydney, Australia) and were used without further purification. NMR solvents were purchased from Sigma-Aldrich and were used without further purification. Anhydrous dichloromethane, was obtained from a LC Technology Solutions Inc. SP-1 StandAlone Solvent Purification System. Analytical thin-layer chromatography (TLC) was performed using Merck aluminium backed silica gel 60 F254 (0.2 mm) plates, which were visualised with potassium permanganate or Hanessian’s stains. Flash column chromatography was performed using Merck Kieselgel 60 (230-400 mesh) silica gel, with the eluent mixture reported as the volume:volume ratio.

*Triolein-d_101_*

To a solution of oleic acid-d_33_ (3.95 g, 12.55 mmol)^1^ and glycerol-d_5_ (0.4 g, 4.12 mmol) in anhydrous dichloromethane (20 mL) at room temperature was added 4-dimethylaminopyridine (0.75 g, 6.17 mmol) and a solution of *N*,*N*′-dicyclohexylcarbodiimide (2.8 g, 13.58 mmol) in dichloromethane (10 mL) over the course of approximately 1 hour. The mixture was stirred for 24 hours before the suspension was filtered through a pad of Celite and the filtrate concentrated under reduced pressure. The crude residue was purified by flash column chromatography using diethyl ether, hexane (0:1 to 1:9) as an eluent to obtain the title compound (3 g, 75%) as a faint yellow liquid,

*R*_f_ = 0.4 (1:19 diethyl ether, hexane. Hanessian’s); ^1^H NMR (400 MHz, CDCl_3_) δ 5.32 (3H, s), 2.33-2.24 (residual, 3H, m), 1.99-1.94 (residual), 1.63-1.53 (residual), 1.34-1.18 (residual), 0.91-0.86 ppm; ^2^H NMR (61.4 MHz, CDCl_3_) δ 5.38 (4D, bs), 4.27 (4D, bs), 2.30 (4D, bs), 1.96 (12D, bs), 1.58 (6D, s), 1.36-01.07 (60D, bm), 0.83 (9D, s) ppm; ^13^C{^1^H, ^2^H} NMR (101 MHz, CDCl_3_) δ 173.5, 173.0, 129.8, 129.7, 129.6, 129.6, 68.5, 61.6, 33.8, 33.7, 33.5, 33.4, 30.6, 28.8, 28.6, 28.3, 28.1, 28.0, 27.9, 26.3, 26.3, 26.3, 26.2, 24.0, 24.0, 23.9, 21.5, 13.1 ppm; LRMS (ESI^+^) [M+Na]^+^ 91.4%D, 0.2% d_101_, 4.4% d_100_, 12.6% d_99_, 15.4% d_98_, 15.7% d_97_, 12.0% d_96_, 9.3% d_95_, 5.9% d_94_, 4.6% d_93_, 3.5% d_92_, 2.4% d_91_, 2.5% d_90_, 2.2% d_89_, 1.8% d_88_, 2.1% d_87_, 1.4% d_86_, 0.9% d_85_, 0.9% d_84_, 0.3% d_83_, 0.5% d_82_, 0.4% d_81_, 0.3% d_80_, 0.9% d_79_.

| 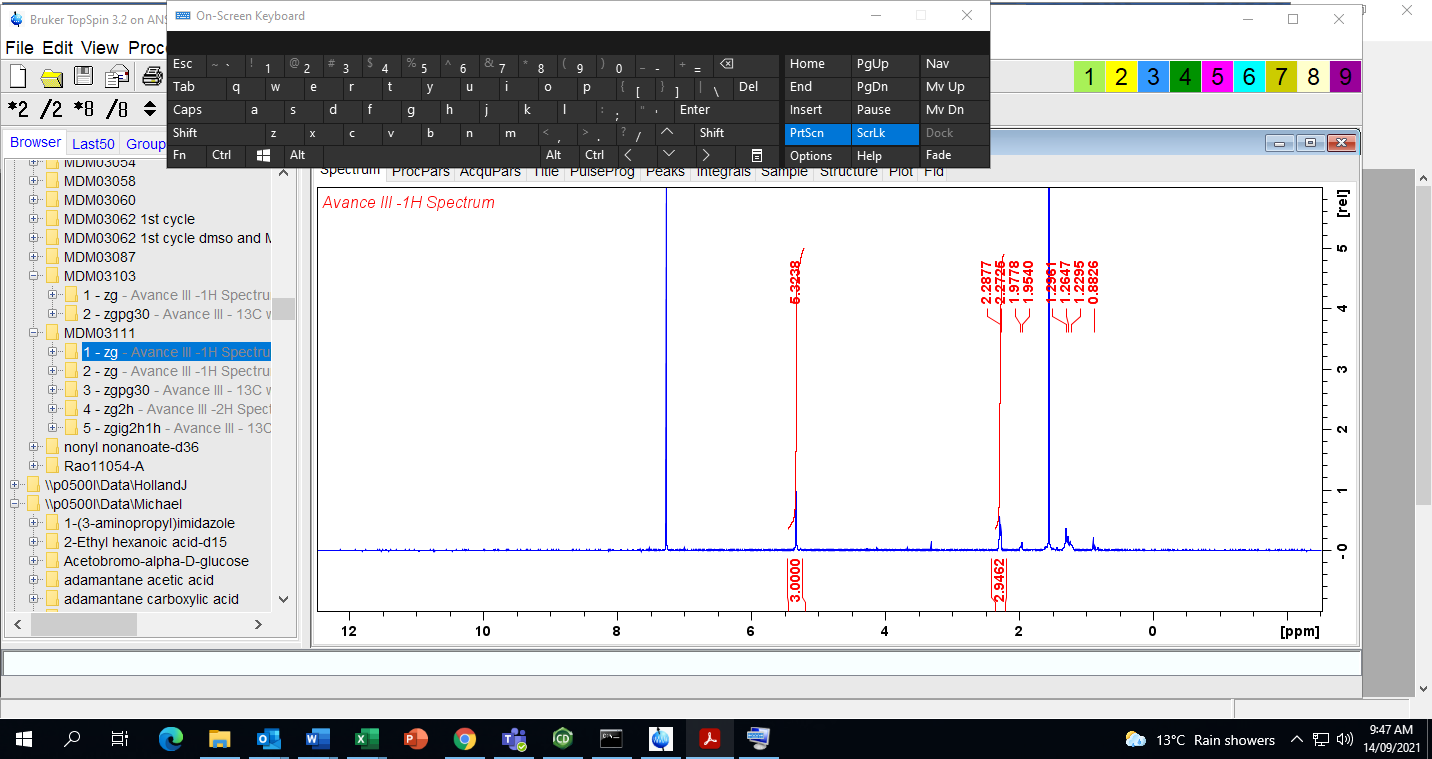 |
| --- |
| **Figure b.** ^1^H NMR (400 MHz, CDCl_3_): Triolein-d_101_. Residual protons observed. Alkene protons calibrated to 3. Suggests back-exchange has occurred at the alpha positions. Singlet at 1.56 ppm corresponds to water. |

| 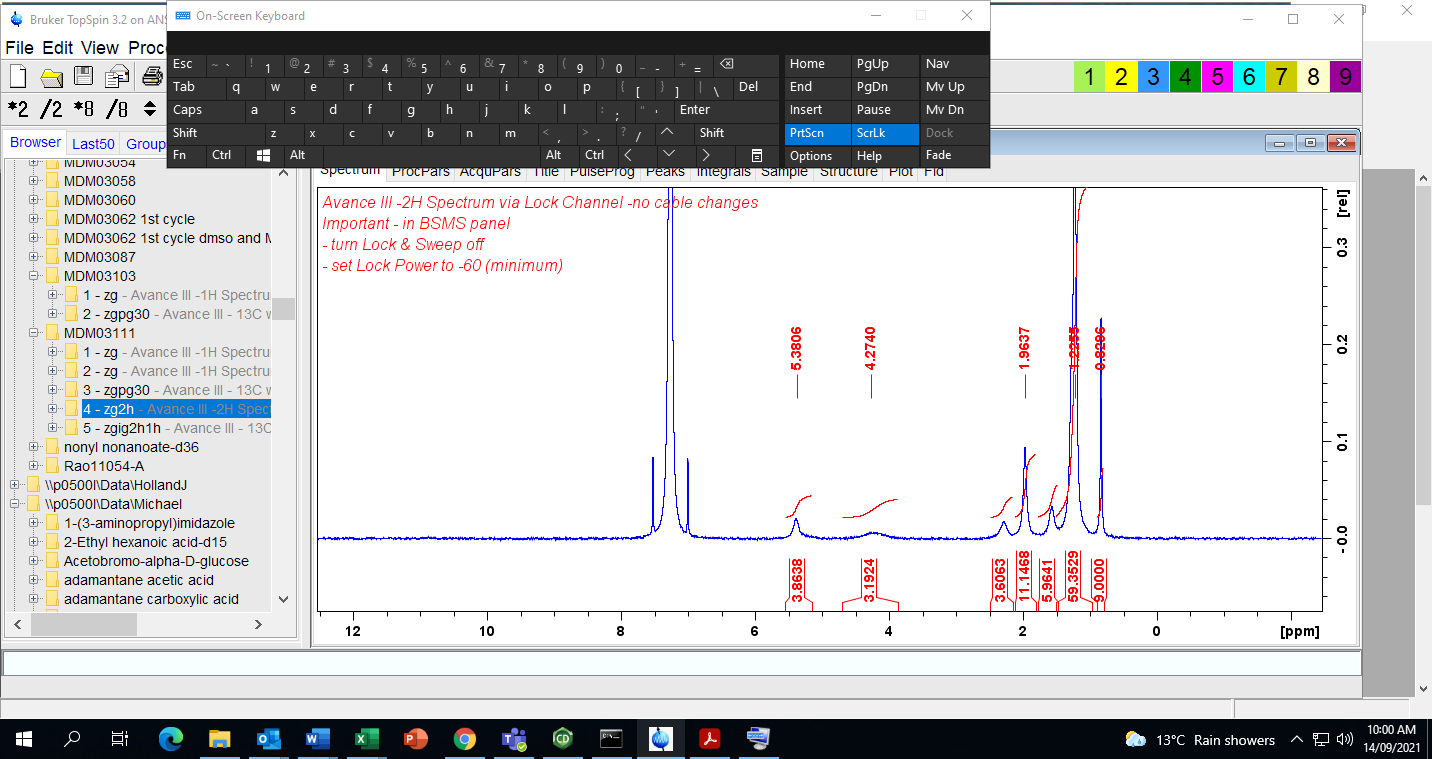 |
| --- |
| **Figure c.** ^2^H NMR (61 MHz, CDCl_3_): Triolein-d_101_. Lower than expected integration of the alpha position suggests back-exchange at this position. Peak broadening of the multiplet around 4 ppm has resulted in low integration at this position (methylenes of the glycerol unit). |

| 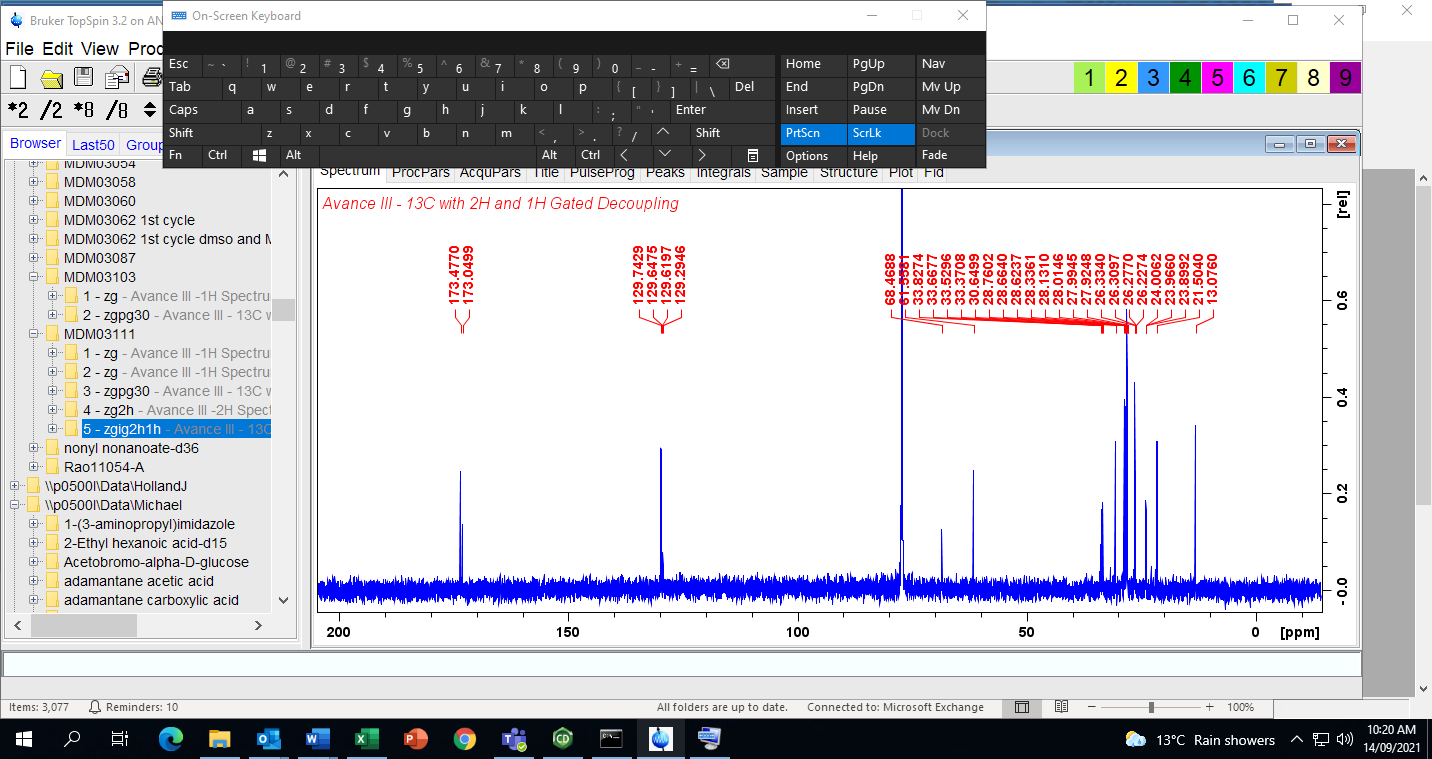 |
| --- |
| **Figure d.** ^13^C{^1^H, ^2^H} NMR (100.6 MHz, CDCl_3_): Triolein-d_101_. Note the alpha carbon (~33 ppm) is split due to the presence of CD_2_ and CDH. |

| 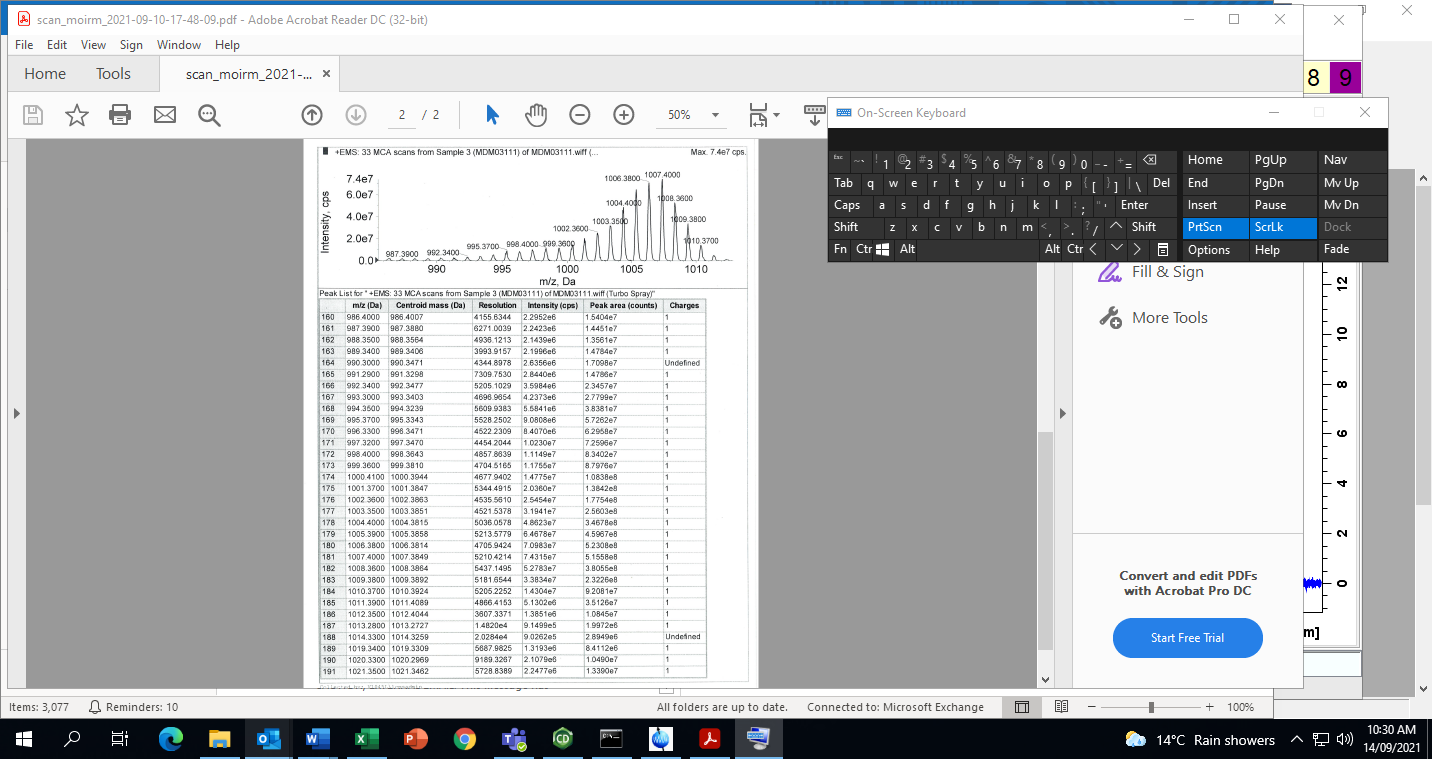 |
| --- |
| **Figure e.** LRMS (ESI) of triolein-d_101_ [M + Na]^+^ observed: 0.2% d_101_, 4.4% d_100_, 12.6% d_99_, 15.4% d_98_, 15.7% d_97_, 12.0% d_96_, 9.3% d_95_, 5.9% d_94_, 4.6% d_93_, 3.5% d_92_, 2.4% d_91_, 2.5% d_90_, 2.2% d_89_, 1.8% d_88_, 2.1% d_87_, 1.4% d_86_, 0.9% d_85_, 0.9% d_84_, 0.3% d_83_, 0.5% d_82_, 0.4% d_81_, 0.3% d_80_, 0.9% d_79_. The overall percentage deuteration of the molecules were calculated by MS using the isotope distribution analysis of the different isotopologues. This was calculated taking into consideration the ^13^C natural abundance, whose contribution was subtracted from the peak area of each M + 1 isotopologue to allow for accurate estimation of the percentage deuteration of each isotopologue.  **References**  [1] Darwish, T.A., Luks, E., Moraes, G., Yepuri, N.R., Holden, P.J., James, M., *J. Label. Compd. Radiopharm.* **2013**, *56*, 520–529.  **Full peak attribution for PalmEA in D triolein (spectra Fig. 2)**: δ (ppm) 6.00 (s, 1H, NH), 3.62-3.50 (m, 2H, C**H_2_**OH), 3.09 (q, *J* = 6.0 Hz, 2H, C**H_2_**NH), 2.80 (t, 1H, OH), 2.04 (t, *J* = 7.4 Hz, 2H, C**H_2_**CO), 1.55 (m, 2H, C**H_2_**CH_2_CO), 1.30-1.26 (m, 24H, 12 CH_2_), 0.86 (t, *J* = 6.8 Hz, 3H, CH_3_). |

**
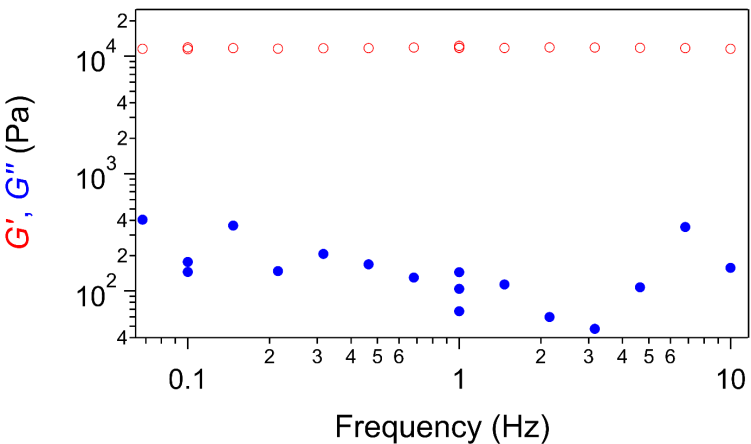
**

**Figure S1.** Variation of the elastic *G’* and viscous *G’’* moduli a function
of temperature of 12-HSA/triolein (*c* = 5 wt. %)

**
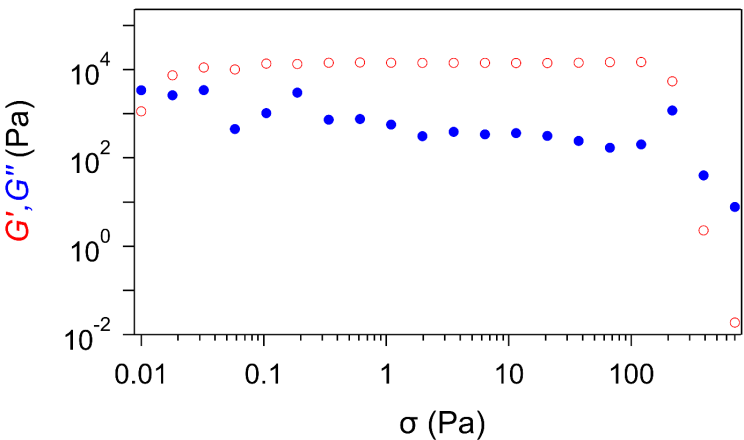
**

**Figure S2.** Variation of the elastic *G’* and viscous *G’’* moduli as function of the applied stress σ for 12‑HSA/triolein oil (*c* = 5 wt. %). The Linear Viscoelastic Region is visible between 0.1 Pa and 70 Pa.

**
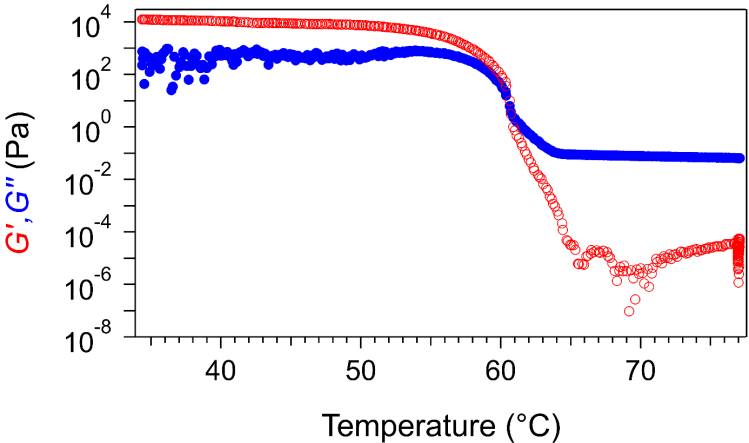
**

**Figure S3.** Variation of *G′* and *G″* as a function of temperature;
12-HSA/triolein (5 wt. %); applied stress: 0.5 Pa; *T* = 25 °C.


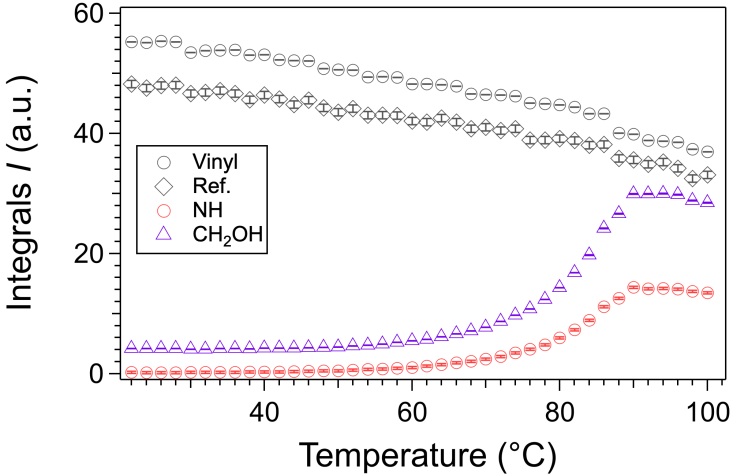


**Figure S4.** Raw integrals vs. temperature for Palm-EA/triolein and for the reference.

**
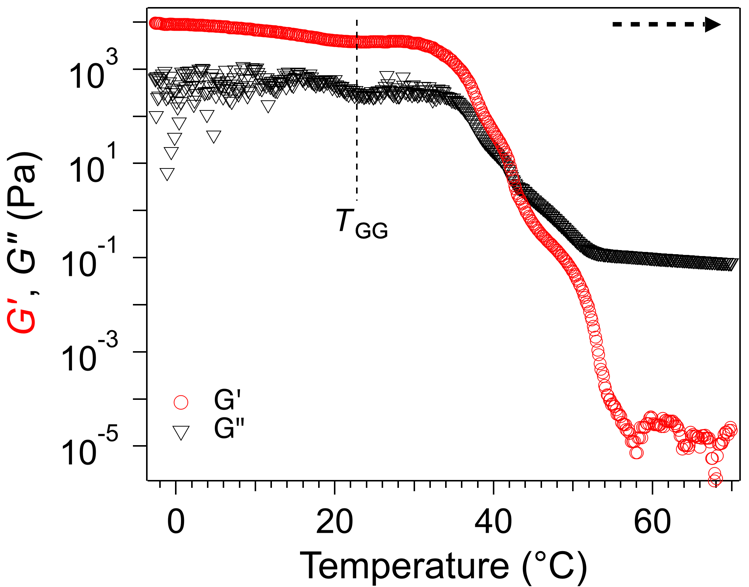
**

**Figure S5.** Variation of *G′* and *G″* as a function of temperature;
Palm-Phe/rapeseed oil (3 wt. %); applied stress: 0.5 Pa; *T* = 25 °C. The decrease of *G′* followed by an increase coincides with the first transition observed by DSC and is attributed to a polymorphic transformation of the solid network.
